# Blockchain and Higher Education Diplomas

**DOI:** 10.3390/ejihpe11010013

**Published:** 2021-02-19

**Authors:** Renato Q. Castro, Manuel Au-Yong-Oliveira

**Affiliations:** 1 FEUP, Faculty of Engineering, University of Porto, Rua Dr. Roberto Frias, 4200-465 Porto, Portugal; up201900049@fe.up.pt or; 2 INESC TEC, GOVCOPP, Department of Economics, Management, Industrial Engineering and Tourism, University of Aveiro, 3810-193 Aveiro, Portugal

**Keywords:** blockchain, higher education, diplomas, certificates, fraud, international students, radical innovation, refugees

## Abstract

Due to added mobility and the increase in international students worldwide, as well as the current problem regarding the counterfeiting of diplomas and the selling of fraudulent certificates, we propose a technological solution. Namely, to ally blockchain technology to higher education certificates and diplomas, to make the process of checking for academic qualifications more facilitated and transparent. Employers of graduates, as well as higher education institutions which evaluate course applicants, would benefit. Perhaps equally as important, students applying for international degree programs would have their lives simplified. There is an increased pressure to ensure the legitimacy and authenticity of certifications and diplomas—and preferably without the current “hassle” of getting diplomas recognized by official entities. New technological advances, with the development of blockchain and smart contracts, with their characteristics of immutability, decentralization, security, traceability, and consensus, may be considered an excellent match to implement a robust and reliable anti-fraud solution to issue digital diplomas. Radical innovations, such as linking blockchain and higher education diplomas, involve significant change and novelty. Linking blockchain and higher education diplomas could potentially positively impact and benefit millions of people worldwide, especially the younger generations. This study involved a literature review and the searching of the Scopus database (refereed publications) for the following concepts: *blockchain* and *diploma*. Existing literature is recent, with most articles (25) published between 2019 and 2020, with 4 in 2018 and only 1 in 2017. This was aligned with our expectations since the development of blockchain utilization outside financial and crypto-assets industries is recent, and it is known as “Blockchain 3.0”. We can additionally affirm that the topic is attracting attention and efforts from researchers worldwide and that some higher education institutions have already implemented ad hoc solutions. As it is, the sector lacks a unified response to the problem of automatic and reliable higher education diploma certification.

## 1. Introduction

The aim of this research is to facilitate access to higher education academic qualifications, including to their quality, in a more transparent fashion. This could be due to the need to eradicate fraudulent practices, or to provide an outlet for individuals without the possibility to transport documentation (e.g., refugees), to be able to capitalize on previous qualifications via the use of a decentralized technological system and platform.

The counterfeiting of diplomas, the selling of fraudulent certificates, and degree mills (organizations that commercialize false diplomas without an associated educational experience) are not a new issue. In the United States, evidence goes back to the Civil War, where the market of fraudulent certificates was a common practice, since 1730. However, recently, the issue is attracting more attention from education institutions, international organizations, and employers [1]. The number of students enrolled in tertiary education worldwide has grown more than 53% between 2006 and 2018, according to data made available by the UNESCO Institute of Statistics. Moreover, the number of tertiary international students has grown steadily in the last 20 years, reaching 5.6 million in 2018 [2] (p. 201). Along with the mentioned expansion of international students in the past two decades looking to acquire higher education degrees abroad and applying for jobs worldwide, there is an increased pressure to ensure the legitimacy and authenticity of certifications and diplomas—and preferably without the current “hassle” (involving both time and money) of getting diplomas recognized by official entities.

Indeed, nowadays, checking for diploma or certification authenticity is a lengthy, manually intensive, and sometimes expensive process. For example, students applying to study abroad may be required to do language translations and international authentications/legalizations (e.g., Hague apostille or other forms of notary services) regarding their original documents as a way to prove their authenticity.

The recent advances of technology with the development of blockchain and smart contracts, with their characteristics of immutability, decentralization, security, traceability, and consensus, may be considered an excellent match to implement a robust and reliable anti-fraud solution to issue digital diplomas [3,4]. In turn, the digital diplomas can be easily assessed and verified by any interested party worldwide, without the need for an intermediary and other certification agents. We consider this possibility to be a radical innovation, in so far as the resources it will save.

Radical innovations—which go “beyond the present technology cycle” [5] (p. 1)—rather than staying “within a technology life cycle” [5] (p. 1), such as linking blockchain and higher education diplomas, involve significant change and novelty. Radical innovations change how we live or go about our day-to-day lives, with a new concept (e.g., the appearance of the Internet), as compared to incremental innovations, which only supply minor improvements to existing products and services (e.g., the Apple iPhone 12 versus the Apple iPhone 11). Linking blockchain and higher education diplomas could potentially positively impact and benefit millions of people worldwide. Additionally, the people most affected will tend to be the younger generations, a segment of the population which are more sensitive to the financial issues involved with official certifications of diplomas.

Another issue which is also relevant is the quality of the diplomas attained. Higher education institutions, and their courses/degrees, receive certifications and are integral parts of a number of national and international rankings. This information should also be automatically appended to the blockchain process, as employers and higher education institutions will want to know: (1) what qualifications applicants have; and (2) how good those qualifications are (relative to other applicants and institutions which concede academic certificates). Additionally, ethical issues may also be added to the process, as this is an increasing concern in society and in the education sector, including in undergraduate medical ethics education [6] and in other such related spheres where the humane component needs to be very present.

In this study, we intend to perform a systematic review of the existing literature regarding blockchain use by educational institutions to understand the current status, especially in the management and issuance of diplomas and certificates, while identifying literature gaps, and pointing out potential future research avenues. One such research avenue regards refugees, who are caught up in “humanitarian crisis settings”, e.g., Jordan and Rwanda—conflict-induced refugee settings [7]. As refugees lack important documentation, which would be essential to their latter well-being and quality of life, we perceive that allying blockchain technology to higher education diplomas in this case will be especially useful, thus perhaps leading to additional global equality. The main issue is well-being, including youth refugee well-being [8].

## 2. Methods

An integrative literature review was conducted by searching the Scopus database (refereed publications) for the following concepts—*blockchain* and diploma*—to identify relevant literature during December 2020. The search included keywords, titles, and abstracts. The query and summary of results are in Table 1.

Existing literature is recent, with most articles (25) published between 2019 and 2020, with 4 in 2018 and only 1 in 2017. This was aligned with our expectations since the development of blockchain utilization outside financial and crypto-assets industries is recent, and it is known as “Blockchain 3.0” [3,9]. In its majority, the documents were conference papers, with 23 documents, followed by five journal articles and one conference review, and one short survey. English written documents were dominant with 29 occurrences. The remaining document was written in Spanish. The Institute of Electrical and Electronics Engineers was the main publisher with 12 documents identified.

Next, to select the documents for review, the query results were downloaded in csv format for further analysis in an Excel spreadsheet. Following this, documents were ranked in descending order by the number of citations and had their titles and abstracts read to identify relevant literature. After that step, a total of 15 documents (two journal articles and thirteen conference papers) remained and were considered for a complete reading. 

## 3. Literature Review

This section will present our integrative literature review, which synthesizes and presents a summary of articles read and the main concepts and contributions. We identified authors from distinct parts of the world and different nationalities, such as China, Taiwan, Vietnam, Portugal, Switzerland, Italy, and others. Therefore, we can affirm that the subject is attracting attention and efforts from researchers worldwide. Moreover, we aimed to indicate the current state of the research for implementing blockchain in diploma issuing and verification. Three documents were discarded after reading. 

**Figure 1:**
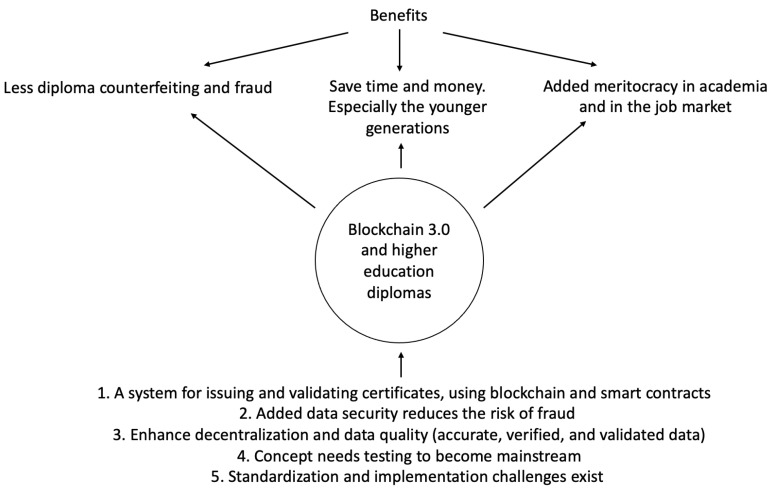
A visual depiction of the research topic.

Figure 1 shows a visual depiction of the research topic. The benefits of uniting blockchain technology with higher education diplomas are represented in Figure 1. The foundations of the future of this system are also portrayed in Figure 1.

The benefits of the suggested system—linking blockchain and higher education diplomas, as shown in the center of Figure 1—are listed at the top of Figure 1: less diploma counterfeiting and fraud (due to decentralized management); save time and money—especially true for the more fragile younger generations; added meritocracy in academia and in the job market (as real qualifications are accessed for processing by higher education institutions and firms). Figure 1 goes on to list what is involved, namely: a system for issuing and validating certificates, using blockchain and smart contracts; added data security reduces the risk of fraud; enhanced decentralization and data quality (accurate, verified, and validated data); note, however, that the concept needs testing to become mainstream; standardization and implementation challenges exist.

A bibliometric analysis was performed with the results obtained (including a total of 30 articles, from the Scopus database search, in the timeframe 2017–2020—please see Appendix A). In this paper we adopted the statistical tool R, executed through RStudio (an integrated development environment for R) and using the bibliometrix [10] package to analyze the information. An interesting analysis is to see how the keywords presented in the article are evolving over time. Table 2 shows the top three keywords and their number of occurrences in each year.

The reviewed articles and papers are summarized and presented in Table 3 (including main objective, contribution, and considerations).

We noticed a recent and growing interest in the topic of Blockchain for Higher Education in recent years, therefore confirming the relevance of the discussion.

A descriptive analysis obtained through the bibliometrix *summary* function is given in Appendix A, for further information.

**Figure 2:**
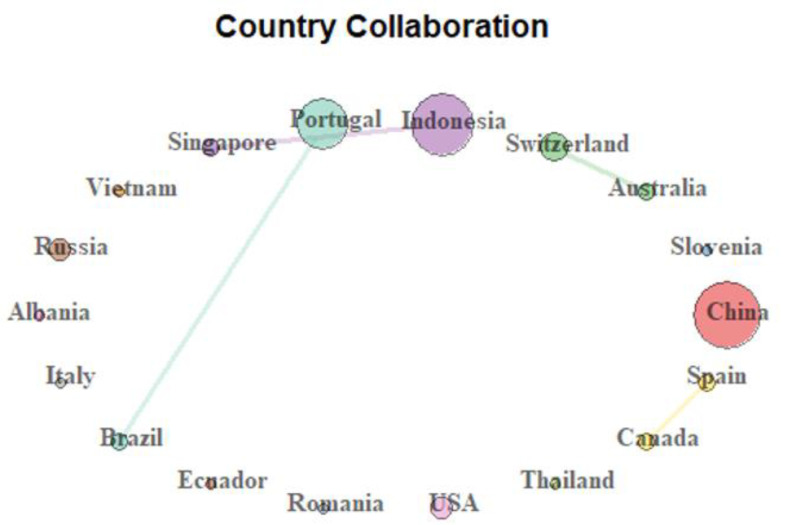
Country collaboration network representation among researchers.

In addition, we searched for authors’ (nationality and) cross-country collaboration (according to the bibliometric analysis) to understand what the current level of research integration regarding the topic is. The results indicate almost no cross-country collaboration except for studies from Indonesia–Singapore, Portugal–Brazil, Switzerland–Australia, and Canada–Spain. Figure 2 shows a graphical representation of the country collaboration network.

In Figure 2, we may see different-sized and different-colored circles, according to the existing country collaborations. There are also (the same) colored lines linking and indicating collaborations. For example, Indonesia and Singapore collaborate (purple circles and a purple line), as does Portugal with Brazil (pink circles and a pink line); again, Switzerland and Australia also collaborate (green circles and a green line); and, finally, Spain collaborates with Canada (pink circles and a pink line). Additionally, China has a large red circle indicating that a number of researchers from China are collaborating together on the topic.

### 3.1. Main Concepts/Contributions

The following description aims to set the scene for the case. One of the authors works at a major Portuguese university and was, until very recently, a committee member on one of the doctoral programs, which required candidates to submit a Hague apostille certification (a form of certificate of authenticity that ensures a public document was issued by an authorized institution, therefore abolishing the need of legalization of such documents abroad) upon application. Of note is that a significant number of international candidates did not know what this was and thus failed to submit it and subsequently were not admitted to the doctoral degree. Moreover, this was a great disappointment, and in some cases, plans had been made for international study and travel. An alternative, decentralized, “watertight” (in so far as intermediate suppliers of information would not be allowed to tamper with the automatically appended information) blockchain solution would make the Hague apostille unnecessary and thus save time, resources, and even a lot of “heartbreak”.

Additional concerns are with fraud. To understand the extent of the problem, the global market size of certificate fraud is estimated at 2 billion USD, according to National Student Clearinghouse, a non-profit organization based in the United States [21]. The costs of fraudulent diplomas may range from USD 350 for a higher education degree to more than USD 4000 for a doctoral degree [22].

Blockchain and its characteristics, in particular the immutability of the transactions, can be seen as being pivotal to the implementation of digital certificates and diplomas in a secure, decentralized, and anti-forgery environment. Thus, it may provide a decisive contribution to the eradication of the existing problems.

#### 3.1.1. Blockchain

Blockchain is a radical innovation that has its origins associated with bitcoin cryptocurrency and the underlying technology proposed by Satoshi Nakamoto in 2008 [23,24]. From the beginning, the technology was associated with crypto currencies; this is what is known as Blockchain 1.0. The introduction of smart contracts represents the surge of Blockchain 2.0, with the development of a new set of applications in financial areas. With the growing interest of several other businesses and industries, mainly because of blockchain’s essential characteristics of decentralization, immutability, and transparency, many solutions are being developed, and we thus enter the Blockchain 3.0 phase [4]. 

Among the industries that may benefit from the novel technology, Higher Education is one that has tremendous opportunities, where the need for document authenticity, transparency, and trust, encounter in blockchain characteristics a great match [4].

In simple and straightforward terms, blockchain can be understood as a distributed database connected in a decentralized manner [3,15]. It is composed of blocks grouped in a transaction. The blocks are cryptographed and linked together to form the blockchain. Each block holds a hash pointing to its predecessor. Using a consensus mechanism, new blocks are validated and linked to the chain [3].

There are three types of blockchain—public, private, or consortium-based [15]—and its access can be permissioned (e.g., an entity regulates the access) or permissionless (where anyone can join) [16].

Smart contracts can be defined as clauses that can be described using a programming language and executed by a computer and were initially proposed in the 1990s. Ethereum introduced smart contracts in its core, making blockchain programmable and, therefore, allowing developers to write a diverse set of applications [3]. Additionally, smart contracts can be seen as an object with the state, attributes, and functions or methods that can be invoked to change states, call other functions or other smart contracts [17].

#### 3.1.2. Blockcerts or Smart Contracts

Blockcerts was designed by Massachusetts Institute of Technology (MIT) Media Lab and further developed by Learning Machine, and now Hyland Credentials. It comprises open-source libraries, components, and applications to issue and verify credentials and build on the bitcoin blockchain [24]. It is considered the first significant case of storing hash certificates and aims to be an open standard for credential management on blockchain [12].

Blockcerts offer a simple way to issue a certificate with the required information and are signed using the issuer’s private key. A hash is generated from a private key and certificate and stored in blockchain informing to whom it was issued. Certificates can be generated in batch for efficiency reasons [15].

Using a mobile app, the Blockcerts Wallet, the receiver (e.g., the student), can easily access his/her certificates and share with whomever necessary. Blockcerts transaction sizes are fixed, and costs to issue certificates are a function of the transaction fee, that is by default 0.0006 bitcoins (approximately 12 EUR, at the time this article was written) [16].

Blockcerts first appearance was in 2017 in a pilot with 111 cohort graduates of MIT [25]. Since then, it has been proposed, tested, and used in several other initiatives, like in South Ural State University [15], University of Rome “Tor Vergata” [17], and University Fernando Pessoa [13].

Nevertheless, Blockcerts is not the only promising open-source solution. For example, in the case of the University of Nicosia, it has developed its own solution based on bitcoin and it was the first university to accept tuition fees in cryptocurrency and issue certificates in blockchain [4,26,27].

On other research fronts, there are the proposals and solutions based on Ethereum and smart contracts, like the ones developed at University of Zurich [12], University of Lisbon [16], and Ho Chi Minh City (HCMC) University of Technology in Vietnam [28].

Like bitcoin, Ethereum provides the same characteristics of transparency, security, immutability, and decentralization, albeit with the additional capacity to be programmable through the use of smart contracts.

On the other hand, advocates of the use of bitcoin claim this is a more mature, tested, and due to the higher financial investment spent, may be the better choice [17].

#### 3.1.3. Digital Diploma Issuing and Verification

The initiatives for diploma management (from issuance to verification) using blockchain are not circumscribed to a specific geographic location or group of researchers. It has spread from Asia [3,11,14,15,28], Europe [4,12,13,16,17], and to the Americas [24] as identified in the literature.

The existing process is clearly identified as inefficient, time-consuming, manually intensive, and costly [17]. All this inefficiency brings attention to the issue of certification forgery [3], which is a significant flaw in the system and affects society in several ways [16]. Surveys indicate relevant numbers of quality issues with certification and diploma information presented in job applications (forgery or fraudulent information) [12,16].

Universities may offer some form of verification or rely on other services for this task to minimize the problem. Despite that, such initiatives suffer from a lack of standardization and unification [12].

Blockchain is seen as a potential solution to improve the process, increase transparency, bring added efficiency, achieve decentralization, and consequently reduce diploma fraud. It can also be used to build a global (transnational) certificate validation ecosystem [16]. Its characteristic of immutability can enhance credibility and reduce the risk of information loss [3].

From the Higher Education Institution (HEI) point of view, blockchain issuance and validation solutions may be beneficial, for example, in internationalization programs, joint-degrees, and international student applications, reducing administrative tasks and costly processes. On the other hand, from the students’ point of view, such systems may simplify student tasks to validate received credentials and eliminate unnecessary intermediaries in the process [4].

As it is, the majority of initiatives are still in early phases of development, as prototype or pilot implementations, and there are only a few applications that surpass that stage and have evolved into commercial applications, even generating spin-offs. This is the case of the University of Nicosia, that, since 2017, has been issuing all diplomas on bitcoin using its own developed open source solution [27,29,30,31].

#### 3.1.4. Initiatives

There is a growing interest in applying blockchain in HEI, with particular attention to issuing and verifying diplomas. Although the authors do not intend to compile an extensive list of current initiatives but shed light on the current status of research on the topic, the literature review identified initiatives, ranging from proposals to prototypes and pilot programs spread worldwide. A summary of initiatives is given in Table 4.

#### 3.1.5. Implementation Challenges/Barriers

To fully deploy the benefits proposed by blockchain solutions in diploma management, implementation barriers must be overcome. Reducing technical complexities to operate the system is crucial and needs to be considered in the solutions [12], such as eliminating the need to deal with public-private key-pair generation [17].

Another challenge originates from the immutability characteristic of blockchain, and it relates to an eventual need to correct information, with special attention to the ability to revoke diplomas and credentials [19].

Data privacy and data protection rules (e.g., the General Data Protection Regulation) must be considered for a successful solution. Again, data immutability in the blockchain may impose barriers to comply with, for example, with the “right to be forgotten” [12].

Solutions must also consider social and organizational aspects, and integrate with existing technological solutions, and deal with previously issued certificates and data storage [12].

## 4. Discussion

In this review, we were able to identify the increased interest in blockchain in the educational environment, especially in solutions for the issuance and verification of diplomas and digital certificates. With its characteristics of transparency, decentralization, and immutability, blockchain finds a perfect alignment with students’ needs, educational institutions, and the labor market in general to minimize the problem of forgery of diplomas. Additionally, it allows for the establishing of a reliable and decentralized process, where those who need to validate the veracity of a diploma dispense with intermediaries, with the process occurring in an efficient and low-cost way. However, like any radical innovation, blockchain still needs to overcome some implementation barriers of a technical nature (complexity of the operation, scalability, correction of errors) and lack of “*de facto*” standards and others of a cultural and social nature (with new business models, regulatory issues, resistance to change). Therefore, investment in research in these areas is still necessary.

In regard to applications in academia and in HEI, with the increase in globalization and with evermore students aiming to study abroad, namely also in Portugal, such a system linking academic diplomas to blockchain might even serve as a catalyst for additional travel regarding higher education studies. With aging populations in Europe, more international students being ready and interested in studying abroad (in a facilitated blockchain-aided process) would make up for the diminishing local student populations (European women, as in all developed countries, are having children at increasingly older ages and are also having less children, quite understandably due to the cost and time involved with the rearing of children) and would thus boost a market otherwise condemned to stagnation (or, in the longer term, condemned to a definitive decrease in market size). This issue is thus of paramount importance and standards and other such related processes need to be addressed for the process to become widespread and mainstream, and not only implemented on an ad hoc basis at a few universities worldwide.

Finally, at a time where good jobs are hard to come by, and where the job market is increasingly more competitive as time passes, the system described herein could be a “game changer”, limiting fraud and providing for a genuinely meritocratic environment concerning academic qualifications. People with the best qualifications would be employed, rather than those able to bypass a system which, at present, is in need of radical improvement.

## 5. Conclusion

We have presented an integrative literature review, summarizing articles read, along with the main concepts and contributions. We have shown how distinct parts of the world and different nationalities, such as from China, Taiwan, Vietnam, Portugal, Switzerland, Italy, and others, have been attracted to the subject. We did a bibliometric analysis using R/bibliometrix. In Appendix A, the commands used are included and part of the result of the summary function. A recent and growing interest is noticeable on the topic of Blockchain for Higher Education, therefore confirming the relevance of the discussion, which is seen to be very timely.

Due to the added mobility of citizens—including the increase in international students worldwide, but also regarding the refugee problem—as well as concerning the counterfeiting of diplomas and the selling of fraudulent certificates—we propose to ally blockchain technology to higher education certificates and diplomas. The result would be to make the process of checking for academic qualifications more facilitated, transparent, and reliable—possibly helping students in more domains than initially predicted and even sparing student burnout [32] (due to the uncertainty involved in the current process). In the case of refugees, this actually might present itself as the only option open to them in the absence of documentation and belongings in conflict-induced/war scenarios. Thus, the benefits accrued to this decentralized process would be up and above the simple adding up of the formal certification process and of the costs saved to job and higher education applicants. The real benefit could be immeasurably higher—providing for a more just and equitable world, where harsh turns of events may mean that technology may be the only solution, as in other scenarios [33], with social support provided by higher education being essential [34].

Our contribution lies also in shedding light on the (lack of a) coordinated approach, in Europe, or involving a group of universities, to solve the problem. We have verified the existence of isolated initiatives of each research group, which look only to the specificities of the countries/universities which they are in, with the exception of EduCTX, which speaks of establishing a global platform for European Credit Transfer and Accumulation System (ECTS), since its conception.

By mentioning the refugee problem, we are calling attention to yet another delicate situation.

Additionally, we leave open the issue of mobility from a more global perspective. We have noted that the initiatives tend to be very local or regional (e.g., Europe) in nature. Concerning international mobility, for example in Portugal, with the increase in the number of Brazilian citizens here (as Brazil has close ties and was a former colony of Portugal, where they also speak Portuguese), who depend on notary processes, the recognition of a Brazilian diploma is not automatic, when compared to a European diploma, which makes the discussion of this topic even more appealing. To recognize a single Brazilian diploma may cost as much as 500 EUR and take 3–6 months. For a refugee without a diploma, things are that much more complicated and with a very significant impact. It is time to unite forces, for a better world. Additionally, is anything more important than our education?

## Figures and Tables

**Table 1:** Initial search query in Scopus.

Query	Documents Returned	Period of Publications
TITLE-ABS-KEY (“blockchain” AND “diploma*”)	30	From 2017 to 2020

**Table 2:** Top 3 keyword occurrences by year, obtained with the KeywordGrowth function.

Keyword	2017	2018	2019	2020
Blockchain	1	5	15	20
Higher Education	0	0	2	4
Students	0	1	4	4

**Table 3:** Reviewed documents including main objective, contribution, and considerations.

Reference	Location	Objective	Contribution	Considerations
Cheng, J. C., Lee, N. Y., Chi, C., and Chen, Y. H (2018) [3]	Taiwan	To solve diploma fraud issues through the use of blockchain.	Developed a system for issuing and validating certificates in the article, using blockchain and smart contracts (based on Ethereum). Solution is built around 3 entities (schools or certification units that issue the documents, students and companies that inquire for a certificate, and service provider responsible for system maintenance and operation). Students are granted an e-certificate (QR code) and information that can be used to assess data.	Authors’ proposed design is very simple, and they do not make any considerations of how schools and certification units will join the network, or how to make sure they are valid institutions allowed to issue certificates. Furthermore, authors did not consider how errors and revokes should be done in their design. This is one of the main questions in the area, as once a transaction is recorded in the chain it cannot be updated. Authors conclude that due to the intrinsic characteristics of blockchain, such solutions can bring trust and reduce issues with certificate forgery.
Kamišalić, A., Turkanović, M., Mrdović, S., and Heričko, M. (2019) [4]	Slovenia, Bosnia, and Herzegovina	Analyzes and categorizes existing blockchain initiatives for Higher Education.	Identification, categorization of initiatives, and their comparison to EduCTX.	The authors are responsible for one of the most referred and daring proposals, the EduCTX that aims to be a global platform for managing “digital micro-credentials”, which makes reading their work worthy. The work lists a good number of initiatives, allowing a good overview of the current state of research development, and the authors perform a useful categorization of those initiatives using two different approaches. In addition, a good description of the EduCTX platform is given along with a discussion about the implementation challenges based on their experience. In our view, it is important to have authors discussing implementation challenges and other aspects besides technical attributes. We understand this is essential to increase awareness of decision makers and increase adoption of blockchain solutions.
Duan, B., Zhong, Y., and Liu, D. (2018) [11]	China	Proposes a specific application of a learning outcome blockchain.	Developed a prototype and executed proof of concept to a group of students at Xiangtan University in 2017.	The article brings a different perspective, by modeling a system for outcome-based learning using blockchain, and how the technology can contribute to creating an open learning environment, involving teachers, students, and even employers, and thereby promote continuous improvement of the curriculum and create greater student involvement. It is interesting in so far as it proposes that the student’s approval does not depend only on the teacher, but is based on a consensus algorithm. By using the proposed system, at the end of the course, the students will have a diploma and a rich set of information about the capacity acquired during the course. For researchers interested in the topic of education learning outcome, and how technology can be used in it, we understand it is an important piece of work, well worthy of being read.
Gresch, J., Rodrigues, B., Scheid, E., Kanhere, S. S., & Stiller, B. (2019) [12]	Switzerland	Proposes a blockchain/smart-contracts based system to issue and verify diplomas for the University of Zurich	A customized solution for the University of Zurich. With a simplified model for interacting with, reducing complexity, when compared to other implementations.	Despite being a specific solution, based on the University requirements some of them can be easily generalized to other locations. Important to notice the authors expressed the need to increase awareness of such solution, so employeers could verify the diplomas by themselves. In our view this is a crucial point for any proposed solution to be widely accepted, but this is not explored further in the paper.
Vidal, F., Gouveia, F., and Soares, C. (2019) [13]	Portugal	A proposed blockchain/Blockcerts-based system to issue and verify at University Fernando Pessoa.	A prototype and metrics about transaction times and costs (per diploma issued) on bitcoin blockchain.	It is interesting to notice that the authors decided to estimate some costs associated with issuing diplomas in a bitcoin/Blockcert solution. Having such numbers is important to help decision makers to compare with the existing process. As there is an expectation that the whole process will be more efficient and cheaper, in particular for students, it is relevant to have research including the cost component.
San, A. M., N. Chotikakamthorn, and C. Sathitwiriyawong (2019) [14]	Indonesia	Proposes a blockchain issue and verification credential method to achieve increased data privacy.	A digital certification validation method based on a Merkle Tree to increase data privacy.	The article brings an interesting perspective by proposing a (theoretical) model for credential management and verification at a very granular and modular level. By sharing a credential, the owner can choose which components they want to be included. The model differs from others (e.g., Open Badge) by the use of a Merkle Tree to build the data model of credentials (courses, learning activities, etc.). Moreover, the model is general enough to accommodate different types of credentials in addition to academic degrees. On the other hand, as it is still a theoretical model, we understand that further development is needed, with proofs of concept and how it would be implemented in a simple and intuitive way.
Nikolskaia, K., Snegireva, D., and Minbaleev, A. (2019) [15]	Russia	Develop a prototype blockchain/Blockcerts for diploma validation.	Set of instructions and diagrams to develop using Blockcerts.	Another prototype implementation of diploma verification application built upon Blockcerts. It is focused more on the technical aspects with diagrams and code excerpts. Worthy to note that the authors made available the source code in github, which can be of interest for some researchers.
Serranito, D., Vasconcelos, A., Guerreiro, S., and Correia, M. (2020) [16]	Portugal	Proposes a prototype of a blockchain/smart-contract ecosystem of Higher Education Institutions for certificate validation.	Describes in detail their unique proposal to enable a consortium of institutions in a decentralized manner and testing results achieved.	Besides technical aspects, the work is useful to shed light on various decentralization aspects that need to be considered in similar solutions. As per the authors, this is not an easy task as “Decentralization is hard because it is not natural for today’s system architects and programmers”. We agree with the authors, because even though blockchain is decentralized by default, a poorly designed solution will compromise the full benefit realization. An additional note is that the solution is being developed in the context of a larger initiative named QualiChain (https://qualichain-project.eu/)The source code in github is available, which can be of interest to some researchers.
Capece, G., Ghiron, N. L., and Pasquale, F. (2020) [17]	Italy	Describe technical aspects of the pilot blockchain/Blockcerts solution at the University of “Tor Vergata”.	Compares issuing and verification for existing and pilot solutions and discusses how blockchain can increase trust and efficiency in the process.	Most challenges were technical, due to the complexity and novelty of blockchain. This can be considered as an essential point for further development—the training of technical resources (like developers, researchers) to work with blockchain. Furthermore, the work confirmed that students are willing to accept such innovation.
Meyliana, Chandra, Y. U., Cassandra, C., Surjandy, Eka Widjaja, H. A., Fernando, E., Prabowo, H., and Joseph, C. (2019) [18]	Indonesia	A proposal for a blockchain model for integrating the university value chain.	An integrated model to achieve enhanced data quality (accurate, verified, validated) for Indonesian universities.	Research focuses on Indonesia’s universities’ value chain to propose a conceptual model to manage full student learning paths until certification. The work does not indicate potential issues, like scalability of the solution, how information will be shared, and data-privacy concerns, among others.
Vidal, F. R., Gouveia, F., and Soares, C. (2020) [19]	Portugal	To present an approach to execute corrective actions on the blockchain to revoke credentials.	A model to revoke digital diplomas that do not depend on actions of third-parties.	The work is useful as it presents a good overview of existing approaches and solutions to deal with certificate revoking. This is an important topic for research because due to the immutability feature of blockchain, any need to change data recorded poses a big challenge. Furthermore, by proposing an alternative model that aims to be blockchain agnostic, it opens research avenues for interoperability and the compatibility of solutions.
Taufiq, R., Trisetyarso, A., Meyliana, Kosala, R., Ranti, B., Supangkat, S., and Abdurachman, E. (2019) [20]	Indonesia	Propose a crypto-governance model for handling student documents and diplomas.	A proposal model to implement a crypto-governance model, involving several actors, using a private blockchain network.	The authors bring an additional perspective to the process, involving multiple stakeholders in the university involved in diploma issuance. On the other hand, it is a customized solution for the Indonesian education system that may be not directly applicable to other jurisdictions. As a note, the solution is built on a private blockchain (IBM Hyperledger), in opposition to the majority of other initiatives that are based on public chains (e.g., bitcoin and Ethereum).

**Table 4:** Identified blockchain diploma verification initiatives.

Institution	Country	Status	Underlying Technology
University of Rome “Tor Vergata”	Italy	Pilot	Bitcoin/Blockcerts
Southern Taiwan University of Science and Technology	Taiwan	Prototype	Ethereum
Xiangtan University	China	Pilot	Smart contracts
Bina Nusantara University	Indonesia	Conceptual Model	N/A
University of Zurich	Switzerland	Prototype	Ethereum
University of Lisbon	Portugal	Pilot	Ethereum
HCMC University of Technology	Vietnam	Prototype	Ethereum
University Fernando Pessoa	Portugal	Prototype	Blockcerts/Bitcoin/Ethereum
South Ural State University	Russia	Prototype	Blockcerts
University of Maribor (EduCTX)	Slovenia	Pilot	Ethereum
University of Nicosia	Cyprus	Production	Bitcoin

## Appendix A

To obtain a summary of the bibliometric analysis, the following commands were executed using the RStudio console:

library(dplyr); library(stringr); library(bibliometrix)

my_scopus <- convert2df("scopus.bib", dbsource="scopus",format="bibtex");

results <- biblioAnalysis(my_scopus)

summary(results, k=10, pause=F, width=130)

The main results obtained from the summary command are as follows:

MAIN INFORMATION ABOUT DATA

  Timespan               2017 : 2020

  Sources (Journals, Books, etc.)      28

  Documents              30

  Average years from publication     1.8

  Average citations per documents     2.533

  Average citations per year per doc     0.73

  References              645

DOCUMENT TYPES

  article        5

  conference paper   23

  conference review    1

  short survey     1

AUTHORS

  Authors                 96

  Author Appearances           107

  Authors of single-authored documents   2

  Authors of multi-authored documents   94

AUTHORS’ COLLABORATION

  Single-authored documents   2

  Documents per Author     0.312

  Authors per Document     3.2

  Co-Authors per Documents   3.57

  Collaboration Index       3.36

Annual Scientific Production

  Year   Articles

  2017   1

  2018   4

  2019   13

  2020   12

Annual Percentage Growth Rate 128.9428

Bibliometrix functions used to obtain the top three keywords’ occurrence and their year distribution:

KeywordGrowth(my_scopus, Tag = "ID", sep = ";", top = 3, cdf = TRUE)

Bibliometrix functions used to obtain the authors’ country collaboration network and plot it.

M <- metaTagExtraction(my_scopus, Field = "AU_CO", sep = ";")

NetMatrix <- biblioNetwork(M, analysis = "collaboration", network = "countries", sep = ";")

net=networkPlot(NetMatrix, n = dim(NetMatrix) [1], Title = "Country Collaboration", type = "circle", size=TRUE, remove.multiple=FALSE,labelsize=1.0)

## References

[1] Grolleau G., Lakhal T., Mzoughi N. (2008). An Introduction to the Economics of Fake Degrees. J. Econ. Issues.

[2] OECD (2020). Education at a Glance 2020.

[3] Cheng J.C., Lee N.Y., Chi C., Chen Y.H. Blockchain and Smart Contract for Digital Certificate. Presented at the 4th IEEE International Conference on Applied System Innovation, ICASI.

[4] Kamišalić A., Turkanović M., Mrdović S., Heričko M., Liberona D., Uden L., Sanchez G., Rodriguez-Gonzalez S. (2019). A Preliminary Review of Blockchain-Based Solutions in Higher Education. Proceedings of the 8th International Workshop on Learning Technology for Education Challenges, LTEC 2019.

[5] Foucart R., Li Q.C. (2021). The Role of Technology Standards in Product Innovation: Theory and Evidence from UK Manufacturing Firms. Res. Policy.

[6] Shamim M.S., Torda A., Baig L.A., Zubairi N., Balasooriya C. (2021). Systematic Development and Refinement of a Contextually Relevant Strategy for Undergraduate Medical Ethics Education: A Qualitative Study. BMC Med. Educ..

[7] de Laat S., Wahoush O., Jaber R., Khater W., Musoni E., Abu Siam I., Schwartz L., Humanitarian Health Ethics Research Group (2021). A Case Analysis of Partnered Research on Palliative Care for Refugees in Jordan and Rwanda. Confl. Health.

[8] Logie C.H., Okumu M., Latif M., Musoke D.K., Lukone S.O., Mwima S., Kyambadde P. (2021). Exploring Resource Scarcity and Contextual Influences on Wellbeing among Young Refugees in Bidi Bidi Refugee Settlement, Uganda: Findings from a Qualitative Study. Confl. Health.

[9] Maesa D.D., Mori P. (2020). Blockchain 3.0 Applications Survey. J. Parallel Distrib. Comput..

[10] Aria M., Cuccurullo C. (2017). Bibliometrix: An R-Tool for Comprehensive Science Mapping Analysis. J. Informetr..

[11] Duan B., Zhong Y., Liu D. Education Application of Blockchain Technology: Learning Outcome and Meta-Diploma. Presented at the 23rd IEEE International Conference on Parallel and Distributed Systems, ICPADS.

[12] Gresch J., Rodrigues B., Scheid E., Kanhere S.S., Stiller B., Abramowicz W., Paschke A. (2019). The Proposal of a Blockchain-Based Architecture for Transparent Certificate Handling. Proceedings of the 21st International Conference on Business Information Systems, BIS 2018.

[13] Vidal F., Gouveia F., Soares C. Analysis of Blockchain Technology for Higher Education. Presented at the 2019 International Conference on Cyber-Enabled Distributed Computing and Knowledge Discovery, CyberC.

[14] San A.M., Chotikakamthorn N., Sathitwiriyawong C. Blockchain-Based Learning Credential Verification System with Recipient Privacy Control. Presented at the 2019 IEEE International Conference on Engineering, Technology and Education, TALE.

[15] Nikolskaia K., Snegireva D., Minbaleev A. Development of the Application for Diploma Authenticity Using the Blockchain Technology. Presented at the 2019 IEEE International Conference “Quality Management, Transport and Information Security, Information Technologies”, IT and QM and IS.

[16] Serranito D., Vasconcelos A., Guerreiro S., Correia M. Blockchain Ecosystem for Verifiable Qualifications. Presented at the 2nd Conference on Blockchain Research and Applications for Innovative Networks and Services, BRAINS.

[17] Capece G., Ghiron N.L., Pasquale F. (2020). Blockchain Technology: Redefining Trust for Digital Certificates. Sustainability.

[18] Meyliana, Chandra Y.U., Cassandra C., Surjandy, Eka Widjaja H.A., Fernando E., Prabowo H., Joseph C. Defying the Certification Diploma Forgery with Blockchain Platform: A Proposed Model. Presented at the 12th International Conference on ICT, Society and Human Beings, ICT 2019, 5th International Conference on Connected Smart Cities, CSC 2019 and the 16th International Conference on Web Based Communities and Social Media, WBC.

[19] Vidal F.R., Gouveia F., Soares C. Revocation Mechanisms for Academic Certificates Stored on a Blockchain. Presented at the 15th Iberian Conference on Information Systems and Technologies, CISTI.

[20] Taufiq R., Trisetyarso A., Meyliana, Kosala R., Ranti B., Supangkat S., Abdurachman E. Robust Crypto-Governance Graduate Document Storage and Fraud Avoidance Certificate in Indonesian Private University. Presented at the 4th International Conference on Information Management and Technology, ICIMTech.

[21] National Student Clearinghouse National Student Clearinghouse and Paradigm, Inc. To Provide Students and Institutions a Secure, Portable, Certified Ediploma. https://www.studentclearinghouse.org/blog/national-student-clearinghouse-and-paradigm-inc-to-provide-students-and-institutions-a-secure-portable-certified-ediploma/.

[22] A Rising Tide of Bogus Degrees. https://www.nytimes.com/2015/05/20/opinion/a-rising-tide-of-bogus-degrees.html.

[23] Nakamoto S. (2008). Bitcoin: A Peer-To-Peer Electronic Cash System. https://bitcoin.org/bitcoin.pdf.

[24] About Blockcerts. https://www.blockcerts.org/about.html.

[25] Durant E., Trachy A. Digital Diploma Debuts at MIT. https://news.mit.edu/2017/mit-debuts-secure-digital-diploma-using-bitcoin-blockchain-technology-1017.

[26] Sharples M., Domingue J. (2016). The Blockchain and Kudos: A Distributed System for Educational Record, Reputation and Reward. Adaptive and Adaptable Learning, Ec-Tel 2016 9891.

[27] Turcu C., Turcu C., Chiuchisan I. (1903). Blockchain and Its Potential in Education. arXiv.

[28] Nguyen D.H., Nguyen-Duc D.N., Huynh-Tuong N., Pham H.A. Cvss: A Blockchainized Certificate Verifying Support System. Presented at the 9th International Symposium on Information and Communication Technology, SoICT.

[29] Fedorova E.P., Skobleva E.I. (2020). Application of Blockchain Technology in Higher Education. Eur. J. Contemp. Educ..

[30] Institute for the Future University of Nicosia Blockchain Certificates (Academic & Others). https://www.unic.ac.cy/iff/blockchain-certificates/.

[31] Block.Co About—Block.Co. https://block.co/about/.

[32] Martos A., Pérez-Fuentes M.d.C., Molero M.d.M., Gázquez J.J., Simón M.d.M., Barragán A.B. (2018). Burnout and engagement in students of health sciences. Eur. J. Investig. Health Psychol. Educ..

[33] Batanero J.M.F., Rodríguez-Martín A. (2017). ICT and functional diversity: Knowledge of the teaching staff. Eur. J. Investig. Health Psychol. Educ..

[34] Fernández-Lasarte O., Ramos-Díaz E., Sáez I.A. (2019). Academic performance, perceived social support and emotional intelligence at the university. Eur. J. Investig. Health Psychol. Educ..

